# Using Narrative Game Design to Increase Children’s Physical Activity: Exploratory Thematic Analysis

**DOI:** 10.2196/16031

**Published:** 2019-11-21

**Authors:** Amy Shirong Lu, Melanie C Green, Debbe Thompson

**Affiliations:** 1 Health Technology Lab College of Arts, Media, and Design, Bouvé College of Health Sciences Northeastern University Boston, MA United States; 2 Department of Communication State University of New York at Buffalo Buffalo, NY United States; 3 Children’s Nutrition Research Center Baylor College of Medicine US Department of Agriculture Houston, TX United States

**Keywords:** narrative, physical activity, active game, children, thematic analysis

## Abstract

**Background:**

Physical activity is crucial for child obesity prevention and intervention. Narratives embedded in active games can increase children’s physical activity.

**Objective:**

Little is known about the narrative characteristics that would motivate children to exercise. We attempted to fill the gaps in understanding regarding narrative design for active video games.

**Methods:**

In this exploratory study, four animated narratives of different genres were professionally generated. Children (N=41) between the ages of 8 and 12 years were interviewed to identify their preferences. Sessions were digitally recorded, transcribed, and analyzed using exploratory thematic analysis.

**Results:**

Findings revealed that the children rated the dystopian science fiction story as their favorite across all weight, race, and gender groups. The physical activity-friendly narrative strategies included virtuous characters, extraordinary character actions, interesting plots, super powers, and engaging cliffhangers. Alternatively, information not related to physical activity, difficult-to-follow plot lines, passive protagonists, and repetitive narrative tropes were less appealing for physical activity.

**Conclusions:**

This research provides preliminary evidence that narratives have characteristics that may increase child physical activity when playing active games. Future empirical studies should verify and test these design principles.

## Introduction

### Background

Childhood obesity is a growing epidemic in the United States and beyond [1]. In 2016, 340 million youth aged 5 to 19 years around the world were overweight or obese [2]. The obesity trend is increasing among many groups, with no ages seeing a decrease [3]. Obesity is a major risk factor for noncommunicable diseases such as cardiovascular disease, diabetes, and several types of cancers [4].

Physical activity (PA) is a critical strategy for child obesity prevention [5]. While it is recommended that children participate in 60 minutes or more of PA every day [6], less than one-quarter meet the recommendation [7]. Most PA interventions have not achieved long-term effects, with lack of access and motivation identified as key challenges [8].

Active video games (AVGs) require body movement to play [9] and may increase PA in an enjoyable manner [10]. With the international popularity of Dance Dance Revolution (Konami), Wii (Nintendo), and Pokémon Go (Niantic), AVG became recognized as an independent genre. In the United States, approximately two-thirds of people play video games daily, and 70% of families have a child who plays video games [11]. All major consoles offer AVG devices [12]. AVG may also encourage PA among children in unsafe neighborhoods lacking safe outdoor alternatives [13].

Many AVGs, though, are perceived to be less enjoyable than sedentary games and are less likely to be played over time [14]. Most gamers do not complete their games [15] as they often lack long-term motivational appeal [16,17]. Novel and interdisciplinary approaches are needed to sustain AVG play [18].

Narratives, or stories, possess unique motivational properties that may encourage increased AVG play [19]. While narratives appear in some health games, few AVGs capable of achieving moderate to vigorous physical activity (MVPA) have incorporated them [20-22]. Recent studies have tested the ability of added narratives to induce PA through AVG play. An exploratory study that interspersed narrative cutscenes (ie, brief, animated narrative video clips) in an existing AVG without a narrative found that children aged 8 to 12 years in the narrative condition had 40% more objectively measured steps than their counterparts who played the nonnarrative version of the same game [23]. Similar results were found in another study involving college students; the narrative increased MVPA by 58% [24].

Both of the aforementioned studies featured narratives that included fantastical elements. For example, the first study used a mystery-themed story of a modern child absorbed into a game world trying to find the way back home [23]. The second featured an ordinary person fending off swarms of monsters to protect the world [24]. However, the question remains as to what narrative elements motivate children to engage in PA. Two exploratory studies provided preliminary insight: actions related to PA should be a central narrative element, a credible PA motivation should be incorporated, and characters should be likeable [25,26]. These studies, however, did not employ a theoretical foundation for analysis and largely consisted of anecdotal observations.

The elusive narrative characteristics that would best motivate PA among children should be explored with the guidance of interdisciplinary theories and in-depth analysis of children’s feedback about the AVG narratives [27]. Here we attempt to fill this gap by exploring narrative design principles.

### Narratives for Health Promotion in an Interactive Technology Age

A narrative is defined as any two or more events arranged in a temporal order [28]. Characters and plot are primary components of narratives. The characters are a major structural property and driving force [29], serving as an internal source of beliefs [30]. As described by social cognitive theory [31,32], characters also function as role models. The plot plays a pivotal role by organizing events into a logically unfolding sequence or temporal order [33].

Narratives have strong potential for health promotion by influencing cognition, affect, and behavior through transportation [34,35]. Transportation is narrative’s unique immersive quality that enables the suspension of disbelief, instills vivid personal experience, and creates deep affection for characters [36].

The advancement of AVG systems has created numerous opportunities for narratives to be better woven with the digital technologies and, in addition, helps amplify the foundational mechanisms of narrative persuasion. For example, the interactive nature of AVGs enhances player engagement with the characters through increased character identification, interpersonal attraction, and parasocial interaction. An appealing plot helps players to go beyond the spectator level to become an active participant and fosters greater engagement with the plot through gameplay. Therefore, an engaging AVG with appealing characters and plots could induce a strong intrinsic motivation to play exergames by reducing cognitive load [37,38] through immersive qualities [39], engendering a positive and powerful arousal and attention [40], enhancing character identification [41], and absorbing players in a story world [42]. The AVG play experience, once integrated with narratives, would internalize reward mechanisms within the players and in turn help foster the perception of exercise through AVG as necessary [19].

### Narratives and Behavior Change Theories

The potential of using narratives can be partly explained by synergies of narrative with several widely used behavioral change theories: theory of planned behavior, social cognitive theory, and self-determination theory. Specifically, the theory of planned behavior [43] posits that a person’s behavior is a function of the intention to perform that behavior, which in turn is a function of the attitude toward performing the behavior, subjective norms, and perceived behavioral control. With their immersive process, the inclusion of narratives in AVGs may make PA seem fun (changing attitudes), may show other characters engaged in PA (which may affect social norms), and can make the behavior seem easy to do (increasing perceived behavioral control), thus inducing a more positive attitude, more positive subjective norms, and greater perceived behavioral control toward performing healthy behaviors [44].

Social cognitive theory highlights observational learning, or vicarious acquisition of knowledge from the social environment, as a primary source of information [31,32]. A narrative AVG has potential through character actions to convey observational learning and useful strategies, model effective style, and demonstrate how to use these strategies, thus enhancing players’ self-efficacy (the belief in one’s capability to achieve different levels of performance), a key construct of social cognitive theory and a similar concept to perceived behavioral control in the theory of planned behavior. The AVG can also offer multiple vicarious experiences to the players to show the consequences of undesirable versus desirable behaviors.

Self-determination theory considers human behavior to be driven by autonomous and controlled motivation [45]. Narrative enjoyment through AVG play can be an intrinsically rewarding activity sought by people independent of extrinsic rewards by providing intriguing internal incentives for audiences who, in the role of characters, feel immersed in the story [46]. Embedding narratives into behavioral change AVGs could potentially promote the development of autonomous motivation to complete the game and adopt the behavior promoted in the game [37].

### Children’s Narrative Preferences Across Media

Little is known about children’s game narrative preferences. Most researchers to date have focused on print [47,48], with some later work conducted examining television [49,50]. Fiction has been recognized as the most popular choice among elementary school–aged children, with around 95% of them reading it [51]. Children have also reported that fiction is easier and more fun to read than other types of stories [52]. Most children’s narrative fiction follows a linear path, with events happening in chronological order [53]. This could be partially explained by the demand to meet children’s developmental needs so that they can understand the plot better.

As for the themes featured in children’s fiction, various scholars have identified different methods to categorize genres. Across genders, intermediate-grade children (aged 5 to 7 years) preferred mystery and adventure stories [54], but children aged 11 to 15 years like fantasy, magic, scary, sorcery, school, romance, and true story genres [55]. Indeed, these themes help to open imaginary worlds for audiences to be transported to a new place.

Recent years have also seen a surge of discoveries about children and young adults’ interest in dystopian science fictions [56-58]. While this preference may be related to the contemporary world and issues brought about by the industrial revolution and global capitalism, the thematic elements in these fictions may also appeal to children and adolescents because they offer strong emotional stimuli and allow them to explore different identities during this particular developmental stage [59].

We attempt to fill gaps in understanding regarding narrative design for AVG. Specifically, we created four different game stories based on narrative and game research with children and adolescents; 41 children between the ages of 8 and 12 years individually provided feedback on each of the four narratives and selected their favorite. We adopted a transcendental phenomenological approach, which aims to uncover the common meaning for multiple individuals of their narrative experience [60]. We then analyzed children’s responses in individual cognitive interviews using an exploratory thematic analysis methodology.

## Methods

### Project Description

This work is part of a larger project systematically exploring the effect of narratives on children’s long-term MVPA through AVG play. We created four professionally made narrative animation clips that convey information about different story arcs to be integrated seamlessly into an existing AVG that had been found to induce MVPA through the Kinect sensor on the Xbox console: Shape Up (Ubisoft) [61]. To ensure the narratives would be engaging and developmentally appropriate, we conducted individual cognitive interviews with 41 children between the ages of 8 to 12 years to gauge their interests and preferences [62].

Our key research questions include: Which of the four narratives would children consider to be the best for motivating them to exercise so that we can develop the narratives further to motivate their long-term MVPA? What are the perceived narrative characteristics that help children to exercise versus characteristics to avoid?

### Sampling and Study Population

We employed a nonprobabilistic, purposive sampling approach to recruit participants from a large, diverse, urban neighborhood in the United States. Many participants came from low-income households. The 8-to-12-year age group was targeted because children younger than 8 years have cognitive limitations in responding to survey questions [63] while children older than 12 years have entered early adolescence and will be subject to many physical, mental, emotional, and social changes that may make their needs and responses different from those of younger children [64]. In addition, without intervention, obese children in this age group are highly likely to become obese young adults [65].

Since children of different weight statuses have different activity patterns when playing the selected AVG [66], we recruited approximately 20 children in each weight group (normal weight vs overweight-obese) to detect potential differences in narrative preferences. Our expectation was that 20 children in each group would be adequate to achieve theoretical saturation (ie, the point at which no new information is attained) [67].

### Narrative Production

We developed four narrative plots to accompany the selected Shape Up game, which requires players to participate in a series of engaging workout exercise sessions involving kickboxing, stomping, squatting, jumping, and so on. Players can see themselves during these exercise sessions thanks to the Kinect sensor. The narrative development process was based on our previous research and experience with children’s narrative engagement [25]. For example, we paid special attention to presenting and justifying the protagonist’s motivation to engage in PA. Each story featured genres we believed to be appealing to children (ie, adventure, mystery, science fiction, and suspense). In accordance with the behavioral theories such as theory of planned behavior, social cognitive theory, and self-determination theory, narratives were created with the goal of encouraging children to play the selected game with increased PA intensity and duration. Each video clip lasted between 3 and 3.5 (mean 3.2) minutes, serving as a story teaser. All clips featured professional-quality animation art and voiceover with distinct styles, and all ended with a cliffhanger that dissolved into a “To be continued...” screen. More specific details about the plot, relevant theoretical concepts in narrative design, and specific design strategies for each of the stories can be found in Table 1. Overall design strategies included the following:

- We ensured that exercise and physical activities are featured throughout the four stories for observational learning while also integrating the physical activities with the narrative’s natural development.
- We had the main characters engage in various types of PA and portrayed both the characters and PA as appealing and fun.
- We created character dialog and plots to elicit emotional reactions and increased enjoyment from children.

We also showed characters encountering and successfully overcoming PA challenges.

**Table 1:** Narrative synopses and design strategies.

Title	Synopsis	Specific design strategies
Food Fight	An adventure/quest fiction about two friends: one of them is a gamer attracted to a mobile game called “Food Fight.” She accidentally spills coffee on the other friend and her phone while volunteering in a nursing home. As they are trying to dry their phones, an accidentally triggered cat-shaped timer sucks the coffee-covered friend into the world of the mobile game and turns her into a game character.	Third-person perspective narration: female player character Main character gender: two girls Main character traits: helpful, considerate, brave, humorous, courageous No antagonist PA^a^ design: character must dodge food attacks (fun and exciting) and engage in PA (jumping and running) to stay alive in game. The gamer character must figure out a way to rescue her friend from the game by engaging in all of these exercises Reward: gamer character receives rewards as she survives each level Modeling: characters demonstrate the exciting aspects of PA Engagement: friendly banter and the fantastical design of the hurdles during the gamified PA acts
#PeepThisSheep	A mystery/suspense fiction about a talented child detective: the detective is lured from a party by a secret note left by someone called “9.” The “9” character tries to recruit the child detective to join a top spy agency because an evil person called Cobalt plans on launching a killer app to dominate the world tomorrow evening. The agency needs the detective’s help. As the detective is preparing to thwart Cobalt’s app launch, a sheep video with a “PeepThisSheep” hashtag becomes wildly popular around the world.	Second-person perspective narration: Gender-neutral player character Main character gender: one boy and “you” Main character traits: intelligent, warm-hearted, brave, spirited One antagonist: Cobalt, who wants world control PA design: detective character suddenly realizes that the video was actually the killer mind-control app and must act fast through engaging in PA (running, fighting, and searching in an interactive and simulated virtual social network) before everything gets out of control Social support: your teammates and your classmates Engagement: player character addresses the children directly and encourages them to exercise and demonstrates that PA is easy and fun and necessary to save the world from the evil Cobalt
Ataraxia	A fantasy/science fiction with the backdrop of a bleak future where a dictator rules the protagonist’s postapocalyptic country: the character’s mother adopted twin babies she found by the roadside and raised them as her own. The family later finds out that the twins do not feel pain and have the power to take pain away from others. The family tries to hide this from the world to protect the twins, but because of the twins’ kind nature and natural inclination to help others, the word gets out.	Second-person perspective narration: Gender-neutral player character Main character gender: a boy and a girl and “you” Main character traits: courageous, nice, compassionate, adventurous One antagonist: evil dictator PA design: the evil dictator discovers this and abducts the twins so he can use their genes to create a force of invincible super soldiers; the character must stop him through various PA engagement (searching, jumping, running, and combat on a future planet) Modeling: the twins keep engaging in fun and exciting PA Social support: the mother figure helps the twins and you to overcome hurdles for PA Engagement: emotional connection with the twins as your “siblings” through vivid details
Star Dust	A mystery/suspense fiction: it begins with two friends’ over-night field trip to an observatory to watch a meteor shower with their classmates and parents, where an eccentric professor greets them. The protagonist discovers an ancient prophecy while inside the observatory: the meteor shower will bring alien matter and wipe out the human race. When the shower begins, everyone outside starts to behave erratically.	Third-person perspective narration: male player character Main character gender: a boy and a girl Main character traits: resourceful, fearless, smart, quick-witted One antagonist: a scientist who keeps the remedy to the alien matter away from everyone PA design: character must figure out how to deal with those who are affected and save the world through engaging in various PA behaviors (dodging infected people, investigating the environment, and searching for a remedy to the alien matter) Engagement: player character needs to solve many puzzles to find out how to save the best friend who may have been infected by the scientist by overcoming many hurdles on the quest

^a^PA: physical activity.

### Data Collection and Analysis

Ethical approval was obtained from the Northeastern University institutional review board. We obtained written informed consent (from parents or guardians) and assent (from children) from all participants. Data were collected between October 2016 and March 2017 at a local community center for families. Demographic information was collected from parents or guardians.

Three research assistants included undergraduate and graduate students majoring in psychology, public health, and communication. Each of them received 25 to 30 hours of extensive training in cognitive interview techniques prior to data collection. They used an interview protocol consisting of 5 structured demographic questions, followed by 8 open-ended cognitive interview questions, and four open-ended follow-up questions for each narrative. Interview questions were as follows:

- Tell me what happened in the story?
- What do you think happens next?
- How do you think the story will end?
- What did you think about the story?
- Was there anything about the story that was hard to understand?
- If you were the writer, would you make any changes to the story?
- Now let’s talk about the characters. What did you think about the characters?
- Which of these characters might be able to help you move around and be more active?

Follow-up questions were asked only if a participant’s response to the initial question did not cover specific topics of interest.

**Figure 1:**
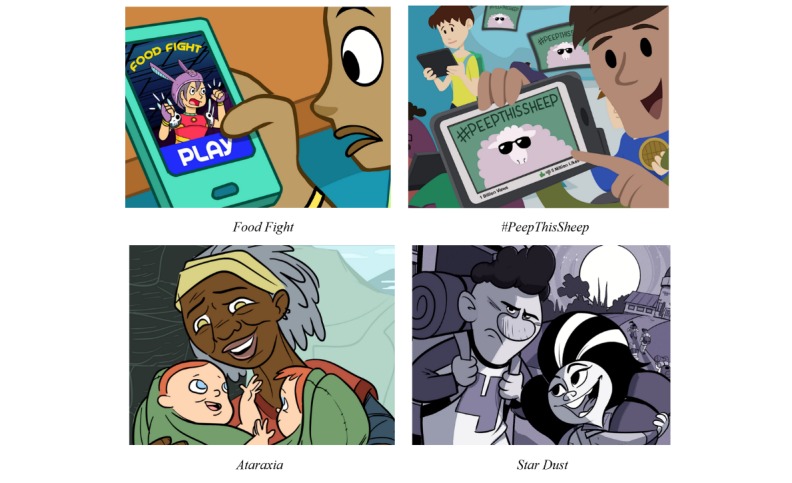
Picture Cards for Narratives.

The four narratives were shown to each child in a random sequence on a laptop to minimize order effects. After each child viewed each of the four narratives and completed the cognitive interviews, the assistants gave them four photo cards (Figure 1) showing the title and a screenshot of each narrative and asked each participant to rank the narratives from most favorite to least favorite by arranging the cards on a table. The assistants documented each child’s narrative rankings (1=most favorite; 4=least favorite) and then asked them the reasons for their rankings. Children were allowed to take a 5-minute break between each story interview, although few did due to their enthusiasm about the animated narratives. Sessions lasted between 50 and 64 minutes. All children answered all questions. Each child received a $25 gift card and they were entered into a drawing to win an OgoDisk set (OgoSport LLC).

The inclusion criteria were between ages 8 and 12 years; able to speak English; and no visual, intellectual, or neurodevelopmental conditions (eg, autism, anxiety disorders). All interviews were conducted in English and digitally recorded. Verbatim responses were transcribed by a professional transcription agency. We then compared the transcripts to recordings for accuracy and revised the transcripts when necessary. To minimize the likelihood of unintentional bias, three coders (undergraduate and graduate students majoring in public health and communication) independently analyzed the transcripts [68]. Coders received around 35 to 40 hours of training for coding qualitative data and cognitive interviews as part of the process of sensitizing concepts [69]. They read each of the 41 transcripts multiple times before the coding process began.

Coding used a hybrid thematic analytic approach. A structured approach was initially used with interview questions providing the framework. Within each question, similar answers were grouped and assigned an emergent code (eg, positive characteristics of a character) [70]. We organized and grouped the codes for each question into higher order codes and then into themes using an open-coding process. The themes were arranged into two categories: features that motivate children to exercise versus features that discourage them from exercising. This strategy was applied to transcripts for all narratives. The coding team met weekly over 3 months with the first author to discuss the codes and organize them into themes within each category. We achieved a general consensus for each category over the meetings and observed theoretical saturation across the interviews.

## Results

### Participant Characteristics

The two weight groups were comparable in multiple characteristics (Table 2). Children of both groups had a moderate degree of experience in playing Kinect games.

**Table 2:** Children’s demographic information (N=41).

Characteristic		Normal weight n=21	Overweight-obese n=20	*P* value
Age in years, mean (SD)		10 (1.6)	10.9 (1.4)	.51
**Gender**		—^b^	—	**.26**
	Boy	10	13	—
	Girl	11	7	—
BMI^a^, kg/m^2^ (SD)		17 (1.9)	25.8 (3.3)	.01
BMI, percentile (SD)		49.7 (26.7)	96.3 (2.1)	.01
**Race, n (%)**		—	—	**.67**
	African American	10 (48)	7 (35)	—
	American Indian/Alaska Native	2 (10)	1 (5)	—
	Asian	1 (5)	2 (10)	—
	Caucasian	5 (24)	5 (25)	—
	Other (mixed)	3 (14)	5 (25)	—
**Parent education, n (%)**		—	—	**.31**
	High school	5 (24)	1 (5)	—
	Technical school	1 (5)	2 (10)	—
	Some college	2 (10)	4 (20)	—
	College graduate	4 (19)	8 (40)	—
	Postgraduate Study	9 (43)	5 (25)	—
**Annual household income (US $)^c^, n (%)**		—	—	**.76**
	<$20,000	6 (29)	4 (21)	—
	$20,000-$39,999	5 (24)	7 (37)	—
	$40,000-$59,999	1 (5)	1 (5)	—
	$60,000-$79,999	3 (14)	3 (16)	—
	$80,000-$99,999	4 (19)	1 (5)	—
	>$100,000	2 (10)	3 (16)	—
**Kinect games (KG) experience**		—	—	—
	How much have you played KG? (1=a little; 5=a l lot)	2 (1.5)	2.6 (1.3)	.15
	How familiar are you with KG? (1=unfamiliar; 5=familiar)	2.3 (1.4)	2.7 (1.6)	.61

^a^BMI: body mass index.

^b^Not Applicable

^c^One family of an overweight-obese child did not provide an answer to this question. Thus, n=19.

### Narrative Preferences

We calculated the preference score distribution and average rank score for each narrative. A lower score indicates higher preference (eg, the favorite story was ranked 1 most favorite, second favorite as 2, and so on). Ataraxia was the top-rated story overall and among all weight, gender, and racial groups. The story takes place in a dystopian future, when a twin brother and sister with the ability to absorb and take away people’s pain have been kidnapped by an evil dictator to create an army of indestructible soldiers. In addition, the interview transcripts indicated children’s negative comments and criticisms were the shortest for Ataraxia compared to the other three options. In general, we did not observe differences across the weight, gender, and racial groups in responses to the narratives; therefore, the ratings of each story were collapsed across all groups (Table 3).

**Table 3:** Children’s narrative preferences.

Title	1 (most favorite)	2	3	4 (least favorite)	Average
Ataraxia	14	16	9	2	1.98
Food Fight	11	5	15	10	2.59
Star Dust	7	11	13	10	2.63
#PeepThisSheep	9	9	4	19	2.80

### Thematic Analyses

The cognitive interview analyses provided rich insights regarding key characteristics of stories (eg, characters and plot) children found effective versus ineffective for motivating them to exercise. Analyses included comments regarding children’s evaluative responses (cognitive and affective) to other narrative characteristics related to our research questions. In comparing feedback, few differences were observed among children of both weight groups. Therefore, their responses were combined. To maintain confidentiality, participant quotes are identified with a randomly generated number between 0 and 300.

#### Responses to the Favorite Narrative

Ataraxia, a science fiction story set in the dystopian future, was selected as the favorite narrative among all weight, gender, and race groups. Most of the themes identified to motivate children to exercise can be found from children’s response to this story. Therefore, they are mentioned here in the order of the frequency observed with a special emphasis on Ataraxia. More specifically, the themes revolved around protagonists, character attributes, and story elements in the order of their observed weight. In the next section, after illustrating the themes with quotes, we connected findings with relevant concepts from health behavior and media psychological theories.

Almost all children identified the twins (ie, the protagonists) as appealing protagonists who would motivate them to exercise. The primary reason usually mentioned was that the activities the twins were performing (eg, leaping from roofs, running after wild boars, or playing with nests of hornets) were fun and exciting.

I think I might want to join them because it looked a little bit fun.

Child 83

The children seemed to have a positive attitude toward PA, which is a crucial determinant of the PA behavioral intention, the strongest predictor of PA behavior according to the theory of planned behavior. This also echoes social cognitive theory’s emphasis on observational learning via role models and self-determination theory’s emphasis on enjoyment in intrinsically motivating children to engage in PA.

The second theme was that the characters had good moral qualities, which echoes the findings that audiences tend to engage with moral characters [71].

Both of them were really brave and really courageous because not only they stayed in the house, but they jumped over...from house to house.

Child 217

They could probably teach me how to be more active and teach me how to be generous that they were in the story.

Child 67

To a certain extent, likeable characters also make social cognitive theory’s observational learning more likely to happen when children identify with the credible characters perceived to have good moral qualities. Similarly, according to self-determination theory, which specifies the basic psychological needs for relatedness to be one of the key precursors for autonomous motivation and engagement, characters perceived to have good moral qualities would foster relatedness by bring children closer to the character who engages in PA. Additionally, such qualities would help to enhance children’s emotional engagement and connections with the characters, thus potentially enhancing immersion.

Last, some overweight-obese children and a few normal weight children mentioned that the twins have special abilities that enable them not to feel pain, which is desirable for exercise.

...so if you get hurt then you can still run around.

Child 163

This is a notable example of narrative’s power to enhance perceived behavioral control, another key influencer of the behavioral intention, which determines behavior according to theory of planned behavior. Enhanced perceived behavioral control could also potentially increase children’s self-efficacy, or confidence in performing a particular behavior according to social cognitive theory. The self-efficacy increase could occur through observational learning or modeling of characters. In addition, self-efficacy can also be enhanced through character identification, when children put themselves into the character’s shoes. This could help children overcome fear of discomfort in performing PA and increase their confidence when they start identifying with the twin characters. Interestingly, quite a few responses suggested strong parasocial interaction [72] with the twins.

...If I was playing with them...like hanging out with them...

Child 97

...If they were my friends, they’d be like cool people to hang out with.

Child 292

These coincided with the positive role model effect and potential emotional affinity and engagement (ie, connection) with the characters. According to self-determination theory, these are important factors helping children to develop autonomous motivation for PA behaviors.

Aside from the twins, other characters also played a role in motivating the participants to exercise. For example, the mother “...seems very nice and she seems like she could give me some confidence” [Child 211].

It seems that the mother character had a positive effect on the subjective norms (ie, children’s beliefs about how people they care about would view their PA behavior according to theory of planned behavior). The mother character’s encouraging gestures and actions would also potentially increase the children’s self-efficacy in PA engagement. The character “I,” who was not shown in the narrative directly, could enhance PA motivation as evidenced by “I get jealous of the video game characters, that they get to do all this running around” [Child 212] or because I want to be “try[ing] to get [my] [family] back” [Child 161], all of which suggested potential emotional engagement.

Interestingly, opinion about the antagonist (evil dictator) was split. While some considered him as a character who would help them to exercise, the action was more of “run[ning] away from him” [Child 158] than running along with him. Many others did not consider an evil character to be trustworthy or motivational.

If this guy is secretly a super villain who wants some mindless soldiers, I don’t think a lot of people would be wanting to do what he does.

Child 2

These responses suggested the importance of positive and engaging narrative character creation, as character with negative qualities may not be as effective at motivating children to perform the desired PA behaviors.

Another theme identified was the story’s immersive characteristics.

You can join the story... That would be cool...[and] you are part of the story.

Child 5

I liked how you were so...like you in the story.

Child 8

I was really intrigued and everything. When it said they [the twins] were gone, I was just like, “Don’t end, don’t end, don’t end,” and then it ended.

Child 44

Many children used the words “interesting,” “cool,” “fun,” and “creative” to describe their overall impression of the narrative. Children also appreciated the amount of detail and explanations offered throughout the plotline.

I think that’s a good thing because there was lots of like plot into the story and lots of details, so I think that was good.

Child 103

[Be]cause it gave reasons for everything...because it explained everything.

Child 162

They also expressed their desire to continue following the story development.

Yeah. I like things that will continue in series. It’ll keep on going and going and going until like the very last.

Child 194

Both children’s affinity for detail and need for additional story development also suggested their high level of narrative involvement with Ataraxia [73], which could potentially lead to increased PA later.

#### Responses to the Remaining Narratives

Additional themes were identified from the other three narratives to explain why the children did not find them as effective exercise motivators. While in general children liked the art styles and plots of these narratives, many used “boring” to describe the characters and plots in the remaining narratives. Some meaningful patterns emerged when we further examined their comments. The results are also focused on protagonists, character attributes, and story elements.

First, there was a lack of interesting or exciting action, which might make the protagonist less attractive. For example, when talking about Food Fight, one child commented, “I think it’s boring because, well the character didn’t actually fight in the game” [Child 5]. Another said that “It’s like—it needs more action” [Child 239]. The same thought was mentioned for #PeepThisSheep as well, “[Be]cause it doesn’t have that much action” [Child 271]. Similarly, another child mentioned that the reason Star Dust was not ranked higher because “...[w]alking, running. It’s all they do” [Child 193]. From a theory of planned behavior framework, these narratives did not seem to help with the participants’ subjective norms by showing PA behaviors as part of the desired action constantly performed by the characters. The narratives failed to provide accessible and attractive behavioral models for children to learn through observation of the character actions according to social cognitive theory. These issues may lead to the failure to instigate autonomous motivation from children, let alone enjoyment, both of which are important in instigating PA behaviors according to self-determination theory.

Second, lack of details about characters and plots seemed to have prevented children from fully understanding and appreciating the narrative developments. For example, when talking about #PeepThisSheep, a child said that the narrative “did not have that much details in it...for [the child] to understand what that means” [Child 216]. Another mentioned adding a bit information to the antagonist.

Maybe just [like] give a little back story on why she wants to release this Peep this Sheep on social media.

Child 2

Similarly, another child suggested offering additional information for how the character in Food Fight was stuck in the phone.

They could give a whole explanation of how she got stuck in the phone.

Child 162

While the exact causal relationship between details and narrative engagement needs additional exploration from an interdisciplinary angle, children’s complaints about the lack of narrative detail suggested their lower level of character appreciation and narrative engagement, which could be less likely translated into PA motivation.

Third, the narrative motifs were perceived to rely on common or overused literary tropes, which might make the plot less interesting. For example, when one child described Star Dust, they said:

Because it was boring. How like—they just go on a camping trip. And then everything just goes bad. And like everybody just starts turning evil. I feel like that’s what happens in most stories.

Child 156

Another described Food Fight by saying:

I’m not saying that Food Fight is bad or anything; it’s just that it could have...been a little bit more creative.

Child 2

Similarly, when describing Cobalt, the antagonist from #PeepThisSheep, one child said:

Cobalt seems like a character I’ve seen before inside a movie.

Child 96

These responses suggested repeated narrative tropes may not bring about the optimal engagement from children to attend to the story development, let alone participate in PA.

In addition, children seemed to take it for granted that there should be a bad guy (antagonist) in the story.

I like that there’s a video and everybody likes to watch it, so a bad guy could hatch a plan.

Child 163, #PeepThisSheep

Cause he’s a scientist and knows about like a lot of stuff and then he’s so evil, but I think evil’s pretty cool.

Child 96, Star Dust

Because like if a story doesn’t have a bad guy, then it’s just going to be boring.

Child 156, Ataraxia

On the other hand, as Food Fight did not feature a true antagonist and shifted the conflicts among the two friends, it did not seem to work for motivating children to exercise.

I have a best friend and me and her, we don’t get into like fights a lot because we know it was gonna ruin our friendship together

Child 83

Because it’s better to say sorry than just get mad at your friends.

Child 173

These responses emphasize the importance of creating interesting antagonists with depth and dimension to maintain children’s interest.

Last, children did not particularly enjoy the spy-themed plot in #PeepThisSheep. More specifically, they seemed to be bothered by how the main character was approached and recruited by “9,” who lured the main character out of a school party with a favorite snack and left a secret note. Quite a few children seemed to be irritated by his actions.

“9” was so secretive; he was kind of creepy.

Child 8

Others equated his behavior to that of the antagonist.

Well, [what] I don’t get the plan is why “9” would be stalking you instead of [Cobalt].

Child 161

Accordingly, they questioned the subsequent plot development.

It was kind of hard to understand how “9” is a stranger to the narrator and then like [the narrator] just ends up going to his basement and...joining...his spy team.

Child 167

## Discussion

### Practical Recomendations

Based on the exploratory thematic analysis, we arranged the two themes identified (features that motivate children to exercise vs features that discourage them to exercise) into preliminary creative design principles. The principles are presented as Dos and Don’ts for narrative creation intended to motivate children to participate in physical activities. The theoretical implications are also discussed. Please note that given the limited sample size and research strategy, all of the recommendations are presented more as exploratory possibilities that need to be empirically verified.

#### Dos

##### Do Create Child Characters With Strong Moral Values

Children identify with and want to model themselves after characters who are good people and who are like them. Both tendencies have been well documented by social learning theory and affective disposition theory researchers [74,75]. While truly bad characters are also needed as antagonists, they may motivate children to be physically active but in a more passive way. For example, the children can escape when the bad guys are threatening their lives. On the other hand, children do not want to see the protagonists fight against each other.

##### Do Involve Extraordinary Actions

Designers should create narratives with characters in constant need of action throughout, or in other words, action-packed narratives. The actions should not be regular mundane routines (eg, walking or running) but something unusual. This concept is closely related to the interactive nature of active games and will also cater well to children’s novelty-seeking tendency in their developmental stage to capture and sustain their attention to the story development.

##### Do Make the Desired Exercise Fun, Interesting, and Integrated Into the Story

Excitement is crucial for inciting children to imitate characters’ actions and ultimately initiate their own PA. Fun and interesting exercise makes PA more desirable and less tedious [76]. When excitement, fun, and interesting exercise are packaged within an active game, children will more likely be intrinsically motivated to exercise over time [77].

##### Do Involve Super Powers When Creating Protagonists and Plots

Fantasies and fictions open up imaginary worlds, enabling children to be transported to the narrative world [78]. Super powers not only could make the physical activities engaging and exciting but also may potentially help remove perceived obstacles for children who are not used to exercise and who would not want to exercise, to increase their self-efficacy.

##### Do Create Immersive Story Plots With Intriguing Cliffhangers

The power of the immersive narrative engagement has been discussed extensively [79]. In addition, suspense is a frequently employed device for maintaining audience interest in stories [80]. Intriguing cliff hangers delivered at the right moment may make children curious about what happens next [81]. They will be eager to continue their narrative engagement through additional exercise behaviors.

#### Don’ts

##### Don’t Omit Information From the Story Development

To ensure that the narratives are organically related to physical activities, relevant information about why the character is engaging in PA should be integrated into the story development. This will help to justify character’s motivation to engage in different types of exercises as a natural progression of plot development, thus making character actions believable and improving the players’ intrinsic motivation to participate in physical activities.

##### Don’t Forget to Provide Reasonable Explanations (With Details) for Why Things Happen

While adults may make the mental bridges as part of their sense-making process to understand and process narratives, the extra effort required may be taxing for children [82] and may reduce their narrative engagement. It is important to assess if a narrative has a well-balanced portion of information given and withheld from children so that their interest in the characters and plot can be constantly piqued but not burdened with too many questions along the way.

##### Don’t Put Protagonists in a Passive Situation to Be Tricked or Watched

An important feature of interactive games is that the players are allowed to be active agents making interactive exchanges within the game to make progress [83]. As a result, while it is reasonable to have some obstacles for the protagonists during the narrative, they should still be given a moderate amount of agency to be in charge of their fate. Putting them in a passive situation (eg, being stalked or watched) may reduce children’s motivation to engage in PA.

##### Don’t Make Boring Stories With a Lot of Ordinary Events or Actions

Children are savvy media consumers [84]. Narrative creators should not underestimate their expectation of innovative stories with unpredictable endings. In addition, they want their imagination to be their guide as they are transported into a fantastical new world instead of getting trapped in the ordinary daily routines.

### Limitations

A limitation of this study is that we relied exclusively on self-report interview responses instead of measuring children’s actual PA behavior in real-world settings. Further studies are needed to discover if narratives produced with these features in mind would indeed lead children to actually play more AVGs. Additionally, the long-term effect of the narrative’s motivation should be validated by children’s play behavior over time. We only used one story from each genre, and it may be hard to pinpoint why exactly children preferred one story over the others due to the different characteristics of the four stories. The animated narratives were not integrated into the game, Shape Up, during this round of data collection, and children did not have the opportunity to play the games after they viewed the videos. Additional studies are needed to explore whether integration of Ataraxia into Shape Up gameplay would motivate children to exercise. Another limitation is that we have a relatively small sample size and only conducted this study in one area of the country; recruited children across many weight, gender, and racial groups from low-income households; and focused primarily on game narrative development, all of which would limit the generalizability of our findings. In other words, while we did not observe differences across the groups, we want to add the caveat that the subgroups were relatively small and could limit our capacity to detect group difference. All of participants, however, seemed eager to participate in such projects and were able to provide insightful responses. Last, we only had one narrative for each genre, which could serve as a confounder. It is difficult to tease apart whether the design strategy or the theme of each narrative was the actual reason for children’s preference. It would be ideal if two or more variations of the narratives within the same theme could be designed and tested with children [85].

### Conclusion

Narratives have immense potential for promoting PA among children through AVGs, but less is known about active game narrative development. Our work here serves as one of the first steps toward a series of systematic inquiries into how to maximize the behavioral potential of narratives for combating childhood obesity. We tried to incorporate existing interdisciplinary psychological and behavioral theories into the narrative development and used direct feedback from our participants to further inform our narrative development process.

A clinically relevant age group (8 to 12 years) was chosen for this project because they are most at risk and closely related to the obesity issue and the age group would be a proper range to intervene. Given the unique persuasive power of narratives, it is also likely that a story developed with this age group in mind would be mostly likely influential among the children. We believe this kind of process will help to create an iterative design methodology for the development of child-friendly narratives that are effective in motivating PA in the future.

From a theoretical perspective, we tried to integrate multiple concepts from behavioral theories (theory of planned behavior, social cognitive theory, self-determination theory, etc) and media psychology (identification, interpersonal attraction, parasocial interaction, etc) into the narrative development stage (see Table 1 for details) and later explored their engagement effect through a close analysis of children’s feedback in the result section. Children seemed to be attracted to positive role models who are engaged in unique forms of PA. Such role models increased their narrative involvement and, to some extent, their self-efficacy. Children also highlighted the way the narratives created autonomous motivation (eg, wanting to be like the characters or spend time with the characters) and increased their subjective norms (eg, important characters encouraging them to exercise).

This research provides preliminary evidence that narratives have characteristics which may increase child PA when playing active games. Appealing features include positive characters, extraordinary actions, interesting plots, super powers, as well as engaging cliffhangers, all of which should be aligned with the exercise motivation. On the other hand, mentally taxing, passive protagonists devoid of agency and hackneyed narrative tropes should be avoided. Future work is needed to verify these findings and examine their effect of PA during gameplay.

## Abbreviations

AVG: active video game
MVPA: moderate to vigorous physical activity
PA: physical activity
